# From development to yield: genetic and molecular regulation of agronomic traits in maize seeds

**DOI:** 10.3389/fpls.2026.1754331

**Published:** 2026-02-04

**Authors:** Babatope Samuel Ajayo, Yubi Huang, Yufeng Hu

**Affiliations:** State Key Laboratory of Crop Gene Resource Exploration and Utilization in Southwest China, Sichuan Agricultural University, Chengdu, China

**Keywords:** agronomic traits, gene regulatory networks, genetic architecture, maize, multi-omics, phytohormonal signaling, protein and starch biosynthesis, seed development

## Abstract

Maize (*Zea mays* L.) plays a critical role in global food security. The challenge of climate pressures and rising population demands emphasizes the urgent need for high-yield, nutrient-rich and resilient cultivars. This review synthesizes the genetic and molecular mechanisms driving maize seed formation, highlighting the development of the embryo, endosperm, and seed coat that influence agronomic traits like kernel size, weight, vigor, and nutritional quality. We investigate how early events, from double fertilization through embryogenesis, endosperm development, and seed coat formation, set the stage for final seed traits. Recent studies, including genome-wide association studies, comparative genomics, candidate-gene analysis, and multi-omics approaches, have shed light on the complex genetic architecture underpinning these seed traits. These studies have identified key regulatory networks involving transcription factors and phytohormonal signals essential for seed development in maize. We also highlight the important molecular pathways that govern starch and protein biosynthesis, alongside nutrient transport processes that are crucial for seed filling. Despite significant advancements, challenges remain in functional validations and integrating multi-omics data across various contexts. Looking ahead, harnessing these insights can drive the breeding of maize cultivars that are resilient, nutrient-dense, and capable of meeting the challenges posed by climate change and rising food demand, ultimately supporting global food security.

## 1 Introduction

Maize (*Zea mays* L.) is central to global food security, providing essential carbohydrates, proteins, and micronutrients for human diets and animal feeds, as well as serving as a raw material for diverse agro-based industries (Dai et al., 2021b; Ajayo et al., 2022). Rising population growth and climate pressures continue to intensify the need for breeding higher-yielding, nutrient-dense, and resilient maize cultivars (Wu et al., 2022; Sethi et al., 2023). Achieving these goals requires a deeper understanding of the genetic and molecular programs that govern seed formation, central determinants of agronomic seed performance and nutritional quality.

Maize seed formation starts with double fertilization and involves the development of the embryo, endosperm, and maternal seed coat. The development of these interconnected tissues determines kernel size, composition, weight, yield, and vigor. The embryo develops to establish shoot and root structures (Doll et al., 2017; Dai et al., 2021b). Meanwhile, the endosperm starts as a coenocytic structure, which cellularizes and differentiates into specialized tissues, including the basal endosperm transfer layer (BETL), starchy endosperm (SE), aleurone (AL), and embryo-surrounding region (ESR). These endosperm tissues facilitates nutrient transport, storage, and mobilization, as well as communication with the embryo (Dai et al., 2021a; Yuan et al., 2024). The maternal seed coat, derived from ovular integuments, protects the seed while enabling biochemical interactions with the embryo and endosperm, essential for seed development and nutrient regulation (Matilla, 2019, 2020; Li et al., 2023a). The coordinated development and interactions of these tissues significantly impact the final agronomic traits of the seeds (Motto et al., 2010; Leroux et al., 2014; Yin et al., 2020; Dai et al., 2022).

Seed traits such as size, weight, starch content, protein composition, and vigor arise from complex genetic architectures involving numerous genes, alleles, and nucleotide variants that interact across environments (Osuman et al., 2022; Sethi et al., 2023; Ndlovu et al., 2024; Qu et al., 2024; Amadu et al., 2025). High-throughput sequencing and phenotyping have accelerated the discovery of loci underlying these traits. Genome-wide association studies (GWAS), combined with expression quantitative trait locus (eQTL) mapping, are revealing regulatory variants that modulate seed developmental gene expression (Li et al., 2022a; Ndlovu et al., 2022; Dai et al., 2024). These insights are now informing marker-assisted selection, genomic selection, and candidate-gene validation strategies aimed at improving seed quality and yield stability.

The integration of comparative genomics, candidate-gene studies, and multi-omics technologies, including genomics, transcriptomics, proteomics, and metabolomics, is rapidly illuminating the molecular networks that link genetic variation to seed phenotypes (Yang et al., 2021b; Gomez-Cano et al., 2024; Huo et al., 2024). These integrative approaches highlight how transcription factors (TFs), phytohormonal signals, and metabolic pathways work together to orchestrate key developmental events, including fertilization, embryogenesis, endosperm development, seed-coat formation, and nutrient transport during grain filling (Yi et al., 2019; Doll et al., 2020; Li et al., 2023a; Peng et al., 2024; Yuan et al., 2024). Recent single-cell transcriptomic studies have further resolved cell type–specific gene expression and functional heterogeneity within the endosperm, revealing previously unrecognized regulatory complexity across BETL, AL, SE, and ESR domains (Yuan et al., 2024). These high-resolution datasets are clarifying gene regulatory networks (GRNs) that integrate transcriptional control, chromatin accessibility, and starch and protein metabolism, influencing developmental programs, nutrient allocation, and storage. Phytohormones, such as auxins, gibberellic acid (GA), cytokinins, and abscisic acid (ABA), interact with these GRNs to coordinate the transition from early development to storage deposition and maturation (Yi et al., 2019; Wu et al., 2022; Yang et al., 2022c; Du et al., 2023; Song et al., 2024).

Despite these rapid progress, substantial challenges remain. Functional validation of candidate genes is still limited, and integrating multi-omics datasets across developmental stages, tissues, genotypes, and environments remains technically demanding. Dynamic metabolic fluxes and the temporal-spatial misalignment of omics datasets further complicate efforts to resolve cell type-specific regulation, especially given the underutilization of single-cell and spatial technologies. Moreover, translating multi-omics discoveries into applied breeding pipelines requires methodological standardization, high-resolution phenotyping, and scalable analytical frameworks capable of associating molecular variation to complex seed traits.

This review synthesizes current understanding of the developmental frameworks that govern early events of maize seed formation, the genetic and molecular networks that shape key agronomic traits, and the metabolic pathways driving storage deposition and seed quality. It also highlights recent multi-omics advances, unresolved challenges, and future opportunities for integrating systems biology with breeding. Together, these efforts will be crucial for developing high-yielding, nutrient-dense, and climate-resilient maize varieties to sustain future global food security.

## 2 Developmental framework of early maize seed formation

### 2.1 Key early seed development programs and tissues in maize

**Figure 1:**
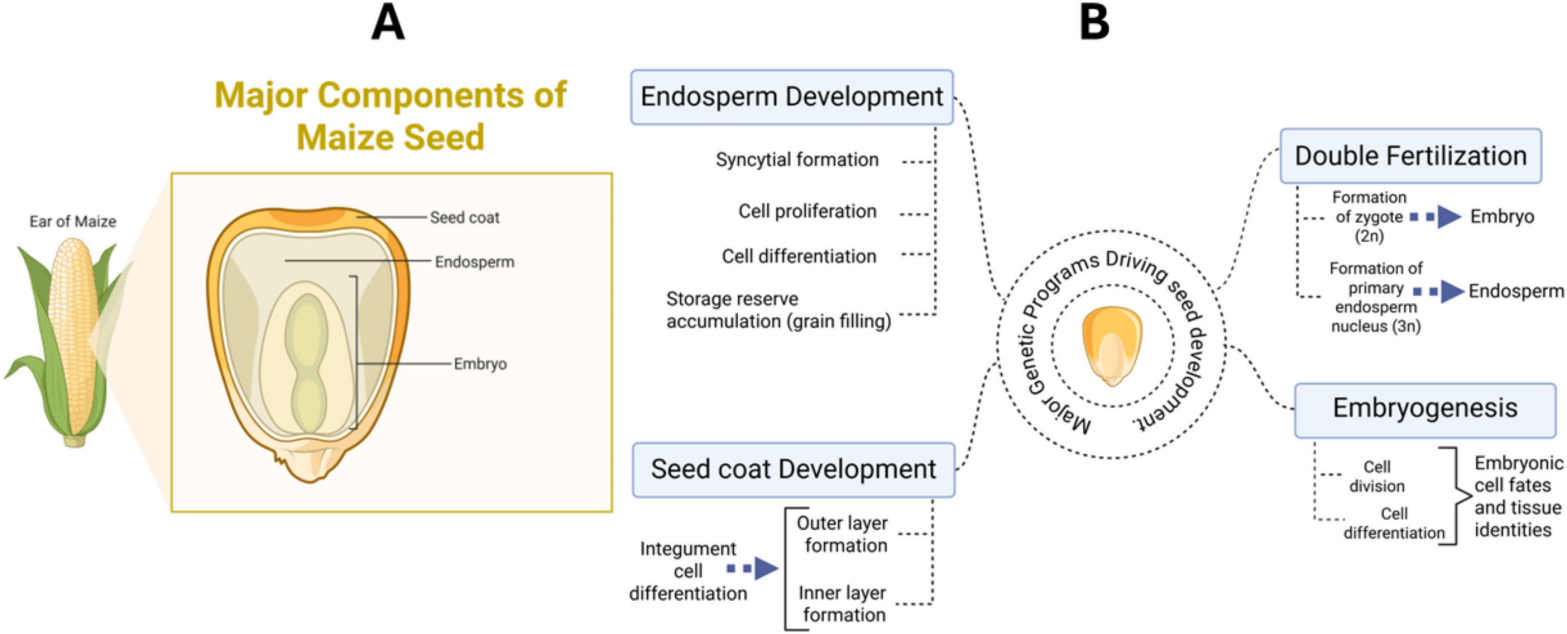
Tissue components of maize seeds and early developmental programs. **(A)** tissue components of maize seeds: a maize seed consists of three primary parts: the embryo and endosperm, both formed through double fertilization, and the seed coat, a protective layer derived from the maternal integuments of the ovary; **(B)** schematic highlights of early developmental programs that precondition agronomic seed traits in maize: (1) double fertilization, which leads to the formation of the zygotic embryo and primary endosperm; (2) embryogenesis, directing the growth and patterning of the embryo to establish its identity and cellular architecture; (3) endosperm development spanning syncytial formation, cellularization and differentiation, and filling, which are critical for storage of nutrients that sustain embryo growth and influence grain size, yield, and nutritional quality; and (4) formation of maternal seed coat from modification of the ovular integuments, which provides protection and coordinates biochemical and signaling exchanges with the embryo and endosperm that ultimately influences the final seed traits. Created in BioRender. Ajayo, S. (2025) https://BioRender.com/dyik1y1.

Maize seed development is a tightly coordinated process that produces three interconnected tissues, the embryo, endosperm, and maternal seed coat (**Figure 1A**), that collectively determine kernel size, composition, vigor, and final yield. These tissues arise from a sequence of early developmental programs, including double fertilization, embryogenesis, endosperm development, and seed coat formation (**Figure 1B**). Understanding the genetic basis and molecular mechanisms governing these early developmental programs is crucial for both seed biology and agricultural applications, as seed traits directly impact yield, nutritional quality, and stress resilience.

Seed formation begins with double fertilization, producing a diploid embryo and a triploid endosperm. Post-fertilization, the embryo undergoes organized divisions and patterning that establish the shoot and root meristems and major organ primordia via embryogenesis (Doll et al., 2017; Dai et al., 2021b). Simultaneously, the endosperm nuclei proliferate to forms a transient syncytium that rapidly cellularizes and differentiates into basal endosperm transfer layer (BETL), starchy endosperm (SE), aleurone (AL), and embryo-surrounding region (ESR). These specialized endosperm tissues mediate nutrient import, storage compound deposition, nutrient signaling, and embryo-endosperm communication, respectively (Dai et al., 2021a; Wu et al., 2022; Yuan et al., 2024). Concurrently, the maternal integuments develop into the seed coat, which provides mechanical protection and regulates water, gas, and nutrient exchange in a manner closely coordinated with endosperm growth (Matilla, 2019; Povilus and Gehring, 2022; Li et al., 2023a). Together, these early developmental processes establish the structural and metabolic frameworks that shapes agronomic seed traits.

### 2.2 Early events in maize seed development

#### 2.2.1 Double fertilization

In maize, double fertilization involves a rapid and highly coordinated delivery of two sperm cells via the pollen tube to the embryo sac. One sperm fertilizes the egg cell to form the diploid zygotic embryo, while the other sperm fuses with the central cell to generate the triploid primary endosperm nucleus. This complex process, which occurs rapidly, require precise pollen tube guidance, gamete recognition, and the subsequent fusion of gametic membranes and nuclear fusion (karyogamy). Pollen tube arrival and gamete fusion occur in less than an hour. This is followed by nuclear fusion in both the egg and central cell, which takes ~3–5 hours, resulting in the formation of zygotic embryo and endosperm nucleus. The zygotic embryo then enters a 13–16 h quiescent phase before its first division, while the primary endosperm nucleus initiates division immediately after nuclear fusion (Takacs, 2012; Dai et al., 2021b; Li et al., 2023b). Flexibility in central cell fusion, which allows the sperm nucleus to merge with either separate polar nuclei or a fused secondary nucleus, facilitates rapid endosperm initiation and development while permitting delayed zygotic division. This adaptability is crucial for controlling and uncoupling embryo and endosperm cell-cycle dynamics (Takacs, 2012; Dai et al., 2021b; Li et al., 2023b).

High-temporal-resolution transcriptomics and advanced imaging have substantially refined our understanding of fertilization-associated signaling. Key molecular players in these processes include signaling peptides derived from the egg apparatus, as well as receptor kinases that facilitate pollen tube guidance and control of tube rupture and sperm discharge (Takacs, 2012; Dai et al., 2021b). Recent high-temporal-resolution RNA sequencing (RNA-seq) has identified embryo sac– and ovule-expressed secreted peptides and receptor-like kinases implicated in pollen tube guidance, tube rupture, and sperm discharge, with tightly staged expression patterns that coincide with fertilization events (Li et al., 2023b). Parallel studies of haploid induction and fertilization bypass have uncovered key membrane and transcriptional regulators, including *MTL*, *BBM*, *DMP*, and *ECS*, that perturb gamete fusion or genome stability, illuminating molecular links between fertilization, early zygotic programs, and genome elimination pathways (Chumakov et al., 2020; Shen et al., 2023). Also, the *GEX2* gene family has been implicated in sperm cell interactions during double fertilization, with mutant allele analyses providing insights into gamete function (Dresselhaus et al., 2016).

Advances in imaging and sample preparation techniques, such as Feulgen staining and optical clearing, have greatly enhanced our ability to visualize fertilization events, including pollen tube entry and early karyogamy. These techniques allow for comprehensive analyses and enable high-throughput screening of fertilization mutants (Kalinowska et al., 2020; Wang et al., 2023b). However, we still have gaps in our knowledge, particularly regarding the specific biochemical components involved in sperm-egg and sperm-central cell membrane fusion and nuclear fusion during karyogamy (Li et al., 2023b; Shen et al., 2023). While some receptor-like kinases related to pollen tube guidance have been found, they have not yet been validated *in vivo* (Li et al., 2023b). Continuous research is needed to better understand the intricate molecular mechanisms and interactions involved in gamete recognition, fusion processes, and the early developmental programs of the zygote (Li et al., 2023b; Shen et al., 2023).

#### 2.2.2 Embryogenesis

Embryo development proceeds through asymmetric cell divisions and conserved morphological stages, starting from the proembryo phase and advancing to formation of the embryonic axis and scutellum. Classical cytology shows that the proembryo stage ends by ~5 days after pollination (DAP), when radial symmetry shifts to bilateral symmetry, leading to scutellum growth and the initiation of the shoot apical meristem. The root meristem develops later, around 7–8 DAP (Schel et al., 2011; Zhou et al., 2017; Ma et al., 2019). Polarity cues emerge before the first zygotic division, influenced by cytoplasmic reorganization in the egg and synergids that affect axis orientation (Schel et al., 2011; Ma et al., 2019).

High-resolution temporal transcriptomes complement these structural studies, identifying hundreds of embryo-specific genes and TFs expressed within hours to days after fertilization. These datasets highlight dynamic activation of auxin biosynthesis, transport, and signaling modules correlated with asymmetric division, axis formation, and early tissue specification. Stage-specific gene expression also reveals early parental genome activation and TF networks underlying embryonic patterning (Ma et al., 2019; Consonni et al., 2022). Key regulators include WOX proteins and LAFL TFs (LEC1, ABI3/Vp1, FUS3, LEC2), which control embryo identity, maturation, and dormancy (Nardmann et al., 2007; Grimault et al., 2015; Lepiniec et al., 2018). Additional regulators such as BBM genes (e.g., *ZmBBML1/2*) promote embryogenic competence (Chen et al., 2014), and conserved modules like the LEC1-MYB118-ZHD5-LEC2-BBM cascade identified in wheat may provide a framework for understanding similar regulatory circuits in maize (Zhao et al., 2023).

Emerging single-cell and spatial transcriptomics are reshaping our understanding of early maize embryogenesis by resolving embryo initiation, cell-type specification, and organogenesis with high spatial and temporal precision. Maize-adapted protocols now enable robust cell-type deconvolution and spatial mapping of regulatory networks that define embryonic domains (Fouquet et al., 2011; Dresselhaus et al., 2016; Yang et al., 2022b). These innovative approaches are revealing cellular heterogeneity, cell-fate trajectories, and spatially resolved TF modules, although fully validated maize-specific TF circuits are still emerging (Yang et al., 2022b). These resources also support reconstruction of putative gene regulatory networks (GRNs) that can be tested by targeted genetics and reporter analyses (Fouquet et al., 2011; Yang et al., 2022b).

Genetic studies further demonstrate that organelle function is essential for embryo progression. Genes like *EMB27* and *DEK48*, which mediate plastid and mitochondrial activities, regulate the transition from zygote to embryo through retrograde signaling; defects in these genes can cause embryo and endosperm arrest (Bayer, 2020; Yang et al., 2022b). Embryo development is closely linked to endosperm cellularization, as mutation affecting RNA processing or organelle function often exhibit nonautonomous effects across seed tissues (Fouquet et al., 2011; Yang et al., 2022b). However, some studies suggest that embryo and endosperm development can be independent, highlighting that embryo development is genetically distinct and influenced by many genetic factors (Li et al., 2022a). Overall, these findings highlight the complex genetic, molecular, and cellular and tissue interactions involved in early maize embryogenesis and seed development.

#### 2.2.3 Endosperm cellularization, differentiation, and growth

Promptly after its formation, the endosperm enters a rapid coenocytic phase, where multiple nuclear divisions occur without cytokinesis, creating syncytium. Endosperm development in maize then transitions to a unique cellularization program. In this process, cell walls form around individual nuclei, first through alveolation followed by a random division of the central vacuole. This pattern is differs from the strict repeated alveolation observed in Arabidopsis, barley, and rice (Mòl et al., 1994; Olsen, 2004, 2020). Recent RNA-seq studies have highlighted the molecular changes that facilitate the transition from multinucleated cells to distinct individual cells. These studies identified important genes related to cell cycle regulation, membrane and cell-wall biosynthesis, and phytohormone pathways that drive this process (Fu et al., 2023; Li et al., 2025). The onset and duration of cellularization vary across genotypes and correlate with endosperm size (Mòl et al., 1994). Genetic and cell-biological studies highlight regulators of mitosis, cytokinesis, and nuclear positioning as essential for this early progression. For example, the kinesin motor VKS1 (ZmKIN11) is required for proper nuclear migration and spindle organization, as *vks1* mutants exhibit cytokinetic defects, reduced cell proliferation, and smaller kernels (Doll, 2019).

As cellularization proceeds, the endosperm rapidly differentiates into specialized cell types: the aleurone layer (AL), starchy endosperm (SE), embryo surrounding region (ESR), and basal endosperm transfer layer (BETL). The AL, a single-cell layer located on the periphery, is rich in proteins and lipids and plays protective and secretive roles, influenced by phytohormone signaling. The central endosperm cells transform into SE, storing starch granules and proteins, while cells near the embryo form the ESR, facilitating communication with the embryo (Yuan et al., 2024; Li et al., 2025). The BETL develops at the endosperm base, where it plays a critical role in nutrient uptake, marked by specific gene activations (Fu et al., 2023; Yuan et al., 2024).

Single-cell transcriptomes reveal deep functional specialization, with BETL and ESR differentiating as early as 4 DAP to support nutrient transport and embryo signaling. In contrast, AL and SE undergo extended differentiation that continues into grain filling stage (Yuan et al., 2024). Key TFs and transporters regulate these processes, with five MADS-box TFs notably controlling transporter genes at the maternal-filial interface (Fu et al., 2023). Different families of TFs, including DOF, NAC, and bZIP, significantly contribute to endosperm differentiation (Fu et al., 2023; Li et al., 2025). Chromatin regulators like histone deacetylase HDA101 are also essential in modifying gene expression to aid transfer cell differentiation (Figueiredo and Sharma, 2025).

Mutagenic studies highlight the complexity of these regulatory networks, showing that disruptions in transcription (e.g., *dek701*), RNA splicing (e.g., *rgh3*), or metabolic regulation (e.g., *smk7a*), can impede cellularization, differentiation, and nutrient storage (Fouquet et al., 2011; Wang et al., 2023b). Phytohormonal regulation also shapes these events, with auxin accumulation between ~5–10 DAP influencing genotype-specific differences in endosperm proliferation, differentiation, gene expression changes, and final kernel size (Lafon-Placette and Köhler, 2014).

The later stages of endosperm development focus on cell expansion and storage of starch and proteins, regulated by various TFs and genes involved in carbohydrate and zein biosynthesis (Zhang et al., 2016b; Feng et al., 2018; Yang et al., 2021a; Ajayo et al., 2022; Chen et al., 2023). These processes involved in endosperm development are interconnected with structural programs and energy signaling that together impact kernel size, composition, and quality through complex cellular, genetic, phytohormonal, and metabolic mechanisms. Although we have gained important insights into maize endosperm development, the biochemical signals and molecular processes that transform syncytial nuclei into distinct cells are still not fully understood.

#### 2.2.4 Maternal seed coat formation and maternal-filial interactions

The integuments of the ovary (maternal tissues) undergo significant transformations to form the protective seed coat (pericarp), which involves expansion, differentiation, and programmed cell death (PCD). These processes are influenced by biochemical interactions with the embryo and endosperm, impacting the final agronomic seed traits (Matilla, 2019, 2020; Li et al., 2023a). High-resolution transcriptomic analyses reveal many early-expressed genes linked to the growth and differentiation of maternal tissues and seed coat patterning (Consonni et al., 2022).

After fertilization, the pericarp goes through several stages: an undeveloped pre-fertilization stage; a post-fertilization thickening stage characterized by cell wall expansion; a rapid expansion stage synchronized with endosperm growth; and a final maturation (strengthening) stage focused on structural reinforcement. These transitions are regulated by specific TFs, cell wall biosynthesis genes, and PCD pathways. Phytohormones, particularly jasmonic acid (JA) and ethylene, along with vacuolar processing enzymes, also play a vital role in pericarp remodeling (Li et al., 2023a).

Simultaneously, the maternal nucellus surrounding the embryo sac is actively eliminated during early endosperm development. A recently discovered NAC–EXPANSIN regulatory module facilitates this nucellar elimination, potentially increasing kernel size and weight, which could enhance yield (Sun et al., 2022). Two distinct PCD domains have been identified in the placento-chalazal of maternal tissues, including an early TUNEL-negative nucellar PCD related to zygotic signaling and endosperm cellularization, and a later TUNEL-positive integumental PCD tied to senescence and defense (Kladnik et al., 2004). Transcriptomic analyses suggest that JA-ethylene pathways and vacuolar processing enzymes are key regulators of these PCD events (Li et al., 2023a).

The maternal-filial interface, made up of specialized tissues, coordinates communication among seed tissues, facilitating nutrient transfer and developmental signaling. Key components include the maternal pericarp, the eliminated nucellus, the placenta–chalaza for vascular connectivity, and the BETL for nutrient uptake. These tissues form a dynamic communication hub that integrates maternal supply with filial demand. Effective interactions among these tissues, mediated by peptides, sugars, and PCD, are crucial for synchronizing seed coat development with endosperm growth, nutrient flow and storage (Bai and Settles, 2015; Matilla, 2019, 2020; Li et al., 2023a).

Regulatory networks controlling maternal-to-filial nutrient transfer have been identified, including five MADS-box TFs that regulate transporter genes for sugar and amino acid movement (Fu et al., 2023). Genomic imprinting adds another layer of complexity, resulting in parent-of-origin-specific gene expression, which significantly affects seed size and weight. For example, the imprinted dosage-effect defective1 (*DED1*) gene encoding an R2R3-MYB transcription factor, exhibits strong paternal expression bias and acts as a temporal regulator of endosperm development by activating early developmental programs while repressing late grain-filling genes. Paternal *DED1* alleles exert pronounced xenia effects on seed size, with reduced or null paternal expression leading to a marked decrease in kernel weight. This highlights genetic conflict and dosage sensitivity at the maternal-filial interface (Dai et al., 2022). Phytohormones, such as JA and ethylene influence maternal PCD (Li et al., 2023a), while auxin and abscisic acid, regulate endosperm transitions and growth (Du et al., 2023; Wang et al., 2023b; Li et al., 2025). Manipulating nucellus elimination or transcriptional regulators can alter kernel size and resource allocation, providing avenues for crop improvement (Sun et al., 2022).

Overall, these findings highlight the coordinated role of maternal signaling, PCD, transcriptional regulation, phytohormonal pathways, and genomic imprinting modulating maternal-tissue remodelling, maternal-filial interactions, nutrient uptake, and endosperm development, crucial for ensuring seed quality and agronomic performance.

### 2.3 How early developmental processes precondition final seed traits

Early maize seed fate is determined by a rapid series of developmental events, from double fertilization through embryogenesis, endosperm cellularization and differentiation, and integument remodeling into the seed coat. These processes collectively establish the structural and metabolic capacity for grain filling. Kernel yield and quality are greatly determined by these early developmental processes that begin shortly after fertilization, within days of pollination (Motto et al., 2010; Sekhon et al., 2014; Dai et al., 2022).

Double fertilization establishes the embryo and endosperm, and early zygotic activation triggers embryogenesis and endosperm coenocytic proliferation. The precise timing and coordination of zygote activation, syncytial endosperm growth, cellularization, and specification of nutrient transport and storage tissues determine the final cell number, transport efficiency, biosynthetic potential, and storage capacity (Motto et al., 2010; Yin et al., 2020; Dai et al., 2022; Yuan et al., 2024). Consequently, early developmental patterning acts as the primary constraint shaping later reserve deposition, seed composition, and overall vigor. Nutrient import efficiency, combined with the biosynthetic potential and storage capacity of specialized endosperm tissues, governs the deposition of starch, zeins, and other reserves. Together, these processes set the fundamental limits on kernel size, weight, and nutritional composition (Yin et al., 2020; Dai et al., 2022; Yuan et al., 2024).

Embryo polarity and organ initiation, driven by auxin signaling, metabolic reprogramming, and early transcriptional regulators, establish the framework for maturation and desiccation tolerance, thereby influencing overall seed viability and vigor (Dolfini et al., 2007). Additionally, embryo–ESR communication modulates endosperm patterning and resource allocation, impacting kernel size, weight, and vigor (Dolfini et al., 2007; Yuan et al., 2024).

Underlying these early developmental transitions are stage-specific genetic programs involving TFs, chromatin modifiers, phytohormones, transporters, and metabolic enzymes (Leroux et al., 2014; Sekhon et al., 2014; Yin et al., 2020; Ahmad et al., 2021; Dai et al., 2022). High-resolution temporal transcriptomes show early waves of seed-specific TFs and chromatin regulators within hours after fertilization, followed by differentiation modules and biosynthetic programs (Sekhon et al., 2014; Dai et al., 2022). Parental dosage and imprinting modulate the pace of early endosperm programs and influence the onset of storage deposition, underscoring how double fertilization exerts regulatory control over endosperm development and storage reserve accumulation (Sekhon et al., 2014; Dai et al., 2022). Mitosis and cytokinesis regulators govern the transition of syncytium-to-cellular endosperm. If these processes are misregulated, it results in fewer endosperm cells and smaller kernels (Motto et al., 2010; Dai et al., 2021b). BETL differentiation depends on chromatin remodeling and specific TF networks that establish an efficient nutrient-transfer interface (Yin et al., 2020). Impaired BETL identity or reduced ZmSWEET4c-mediated hexose transport restricts import into the endosperm, leading to poor seed filling and smaller kernels (Zhang et al., 2016a; Yin et al., 2020). After cellularization and differentiation, the endosperm enters grain filling stage, marked by rapid starch and protein accumulation, and cell expansion (Yin et al., 2020; Dai et al., 2021a). Filling rate and duration relies on the structural capacity of the endosperm, biosynthetic enzyme activation, and sustained maternal nutrient supply (Dolfini et al., 2007; Motto et al., 2010; Zhang et al., 2016a; Yin et al., 2020; Dai et al., 2022). Early gene sequential activation via TFs such as O2, PBF, ZmNKD1,2, and partners, preconfigures endosperm for storage, linking early regulatory networks to final reserve composition (Zhang et al., 2016b; Chen et al., 2023; Wu et al., 2024).

Maternal control further shapes seed outcomes. Reciprocal crosses demonstrate strong maternal influences on kernel size, filling patterns, and early transcriptomes (Yin et al., 2020). Maternal-paternal genome balance modulates the timing of endosperm development transitions, with maternal excess accelerating maturation and paternal excess extending early programs (Leroux et al., 2014). The maternal seed coat development shapes final seed size by combining early heterotic growth with regulated phloem provisioning, epigenetic control, maternal-driven imprinting, demethylation, and PRC2 activity. These processes modulate resource allocation and developmental timing, leading to endosperm expansion and overall kernel growth (Yin et al., 2020; Li et al., 2023a).

Conclusively, early processes influence final seed trait outcomes by establishing the cell number, transport capacity, and the timing and duration of grain filling. Embryo patterning and embryo-endosperm signaling regulate resource allocation, maturation, and desiccation tolerance, thereby influencing vigor. Endosperm cellularization sets sink size; BETL transport capacity determines filling rate; and sequential activation of starch and protein gene networks determines composition. Maternal seed-coat growth and imprinting further modulate developmental pace and nutrient delivery. Together, these coordinated early developmental programs and interactions determine the overall capacity and tempo of provisioning that specify final seed size, weight, nutrient content, and viability.

## 3 Genetic networks governing agronomic seed traits in maize

### 3.1 Genetic architecture underlying agronomic seed trait expression in maize

Seed traits such as size, weight, starch, and protein compositions are primarily determined by complex genetic architecture involving genes, alleles, loci, and nucleotide variants such as SNPs and indels, that influence trait expression across diverse environments. These genetic determinants control seed development programs and accumulation of storage compounds, which are critical for improving maize yield, nutritional value, and stress resilience (Sethi et al., 2023; Ndlovu et al., 2024; Qu et al., 2024; Amadu et al., 2025).

GWAS has uncovered many loci linked to seed traits. In sweet and waxy maize, 49 SNPs (primarily on chromosome 3) and 338 haplotypes were associated with seed size and weight. Among 40 candidate genes identified by the GWAS, *Zm00001d000707* and *Zm00001d044139* showed seed-specific expression, though their functions remain uncharacterized (Qu et al., 2024). Another GWAS revealed a distinct genetic basis controlling seed size, embryo size, and seed-to-embryo ratio. Notably, 11 maize orthologs of *Arabidopsis* embryo-defective genes were linked to these traits, underscoring conserved genetic programs across species (Li et al., 2022a). In two maize populations, integration of GWAS with eQTL mapping identified cis-regulatory variants that modulate expression of key seed developmental genes such as *ZmKL1* (*Zm00001eb158710*). Stacking favorable cis-regulatory variants has been shown to enhance seed size, presenting a practical breeding strategy (Li et al., 2022c).

Under optimal and drought-stressed conditions, GWAS identified 172 stable quantitative trait nucleotides (QTNs) related to maize grain yield, with 77 and 95 QTNs associated with drought and optimal conditions, respectively. Among 43 candidate genes, *ZmEREB60* and *ZmTCP20* were significantly associated with yield under drought (Amadu et al., 2025). Another study under combined terminal drought and heat stress identified four pleiotropic SNPs and 12 candidate genes linked to grain yield, including those with phytohormone-responsive cis-elements, further emphasizing the role of phytohormonal regulation in yield stability (Osuman et al., 2022).

The genetic basis of seed quality traits, such as starch, protein, and lipid content, also reveals polygenic inheritance patterns. Linkage mapping, genomic selection (GS), and GWAS have identified numerous QTLs governing seed composition under nutrient-limited conditions (Ndlovu et al., 2022, 2024; Dai et al., 2024). For example, analysis of amylose content in 305 inbred lines across four environments revealed 16 QTLs through linkage mapping and 17 SNPs through GWAS, with *ZmGALT29A* and *ZmSWEET4a* emerging as candidate genes involved in sugar transport and starch biosynthesis (Dai et al., 2024). In popcorn, GWAS on temperate and tropical populations identified 13 candidate genes associated with zein protein and starch composition. Alpha-zein genes on chromosome 4 were particularly linked to protein content, while starch traits exhibited subtle population-specific variation (Gotardi et al., 2024). Comprehensive analyses of seed compositional traits using near-infrared spectroscopy (NIRS) and GWAS across 501 maize lines identified 72 SNPs associated with 11 seed traits including starch, carbohydrates, lipids, and proteins (Renk et al., 2021). Likewise, micronutrient profiling via GWAS in 244 lines revealed 824 QTLs and 524 candidate genes, such as *ZmHMA3*, a key regulator of cadmium accumulation, and several ABC transporters and MYB TFs associated with micronutrient transport (Chen et al., 2024a). Collectively, these studies reinforce the profound influence of genetic variability on maize grain yield and quality, thereby supporting the use of marker-assisted and genomic selections for seed trait improvement.

### 3.2 Comparative genomics and candidate gene mapping for maize agronomic seed traits

Analyzing maize seed traits through comparative genomics and candidate gene mapping combines genetic mapping techniques to identify QTLs and candidate genes associated with seed quality and yield-related traits (Sethi et al., 2023; Wang et al., 2023a; Tang et al., 2024; Tianxia, 2024). Candidate gene mapping is defined as a method used to identify specific genes that correlate with QTLs that impact desirable agronomic seed traits. This methodology is crucial for unravelling the genetic framework underlying these traits and advancing marker-assisted breeding efforts (Dong et al., 2025).

Recent research has highlighted the effectiveness of integrating methodologies such as meta-QTL analysis, regional association mapping, and GWAS with transcriptomics to identify stable QTLs and candidate genes linked to seed quality and yield traits in maize, such as *Zm00001d004491*, *Zm00001d035206*, *Zm00001d020926*, and *Zm00001d020461* (*qKW7b*) (Li et al., 2016; Gul et al., 2022; Zhang et al., 2022b, 2023; Sethi et al., 2023; Wang et al., 2023a; Chen et al., 2024a; Tang et al., 2024; Zhou and Hong, 2024).

For example, Chen et al. (2024a) conducted a GWAS involving 244 inbred lines, uncovering 842 QTLs tied to essential micronutrients such as zinc and copper, as well as harmful elements like cadmium. They identified key candidate genes, including *ZmHMA3*, which plays a role in cadmium accumulation, and ten transport genes that can inform breeding for improved nutritional quality. Similarly, Tang et al. (2024) used 260 inbred lines to improve kernel quality traits, revealing 23 significant SNPs associated with grain quality and compiling 697 QTLs, which led to the identification of 40 meta-QTLs and 19 functional genes associated with grain quality. This foundational work underpins future maize quality enhancement. Further studies, including those by Gul et al. (2022) and Zhang et al. (2022b), have expanded our understanding of kernel traits and yield. Gul et al. identified the QTL qKWid9, important for kernel width and overall yield. Zhang et al. uncovered 51 stable and 36 pleiotropic SNPs linked to nine yield traits, highlighting agronomic traits critical for breeding strategies. Also, Li et al. (2016) identified and fine mapped qKW7, a major QTL affecting kernel weight and width in maize. They localized qKW7 to a 647 Kb region with multiple genes using backcross populations. Analysis of 627 diverse inbred lines uncovered three QTNs associated with qKW7, all located within the gene *Zm00001d020461*, which encodes an ankyrin protein kinase involved in endosperm development. In a meta-analysis, Zhang et al. (2023) reviewed 30 years of QTL research, pinpointing 23 QTL hotspots relevant for ear traits and offering insights to breeders aiming to target stable QTLs. Wang et al. (2023a) further explored the complex genetic basis of grain yield, identifying 31 consensus regions for kernel size traits and emphasizing the need for validation of candidate genes. These findings illustrate the interconnectedness of GWAS, transcriptomic analysis, and QTL mapping, enhancing breeding strategies, such as marker-assisted and genomic selections, for both yield and quality traits in maize.

While these multiple studies have identified many QTLs, SNPs, and candidate genes linked to important seed traits, discrepancies exist in the specific loci and candidate genes reported. Some studies focus on major QTLs like qKW7 and qKWid9, while others identify numerous minor loci or novel genes. These differences may be due to variations in genetic materials, mapping populations, marker density, environmental conditions, and the statistical models used, all of which affect the detection of loci and prioritization of candidate genes.

Comparative genomics reveals strong conservation of genetic mechanisms underlying seed traits across maize and related cereals, such as rice and wheat. The identification of orthologous genes and syntenic regions offers insights into shared pathways that regulate seed traits, such as size and weight. This presents opportunities to transfer functional insights and utilize genetic resources across different species (Li et al., 2010; Liu et al., 2017; Sethi et al., 2023). Notable examples of conserved regulators include *ZmINCW1* and *GW2*, both of which play central roles in seed development (Liu et al., 2017; Sethi et al., 2023). Functional conservation is further underscored by successful cross-species applications, such as the introduction of the rice *GS5* gene into maize, resulting in improved kernel size (Dong et al., 2022). These findings emphasize the significance of comparative genomics in identifying conserved genetic targets, guiding breeding strategies, and accelerating trait improvement across cereal crops.

Despite shared genetic elements, maize has unique genetic mechanisms and gene duplications that hinder direct comparisons with other species. Notably, duplicated genes in maize that influence seed traits operate through distinct regulatory networks shaped by maize-specific events such as gene duplications and promoter modifications (Li et al., 2010; Corbi et al., 2011; Hufford et al., 2012). While many QTLs and candidate genes have been cataloged, difficulties in accurately mapping and validating genes, especially those related to seed development, remain significant (Ma et al., 2021; Qu et al., 2024; Wang et al., 2024a). The polygenic nature of maize seed traits, compounded by limited environmental replicates in studies, complicates their direct application in breeding programs. Many QTLs exhibit environment-specific effects, further limiting their usefulness (Li et al., 2019). The translation of genetic discoveries into practical breeding gains remains slow due to the complexity of traits, epistatic interactions, and environmental impacts. Additionally, the distinct genetic control of seed subcomponents such as embryo size versus endosperm traits, adds further complexity to breeding efforts (Lan et al., 2018; Li et al., 2022a; Zhang et al., 2025). Ongoing debates about the roles of additive versus epistatic effects, QTL consistency across environments, and effectiveness of genomic selection are further impeding progress in developing precise breeding strategies to improve yield traits (Li et al., 2022a; Zhang et al., 2025).

## 4 Molecular mechanisms and regulatory networks controlling maize agronomic seed traits

Advances in high-throughput sequencing and phenotyping have enabled multi-omics analyses that clarify the genetic and molecular pathways shaping maize seed traits, including yield, nutritional quality, and stress resilience (Chaturvedi et al., 2024; Gedil et al., 2024). Integrated genomics, transcriptomics, proteomics, and metabolomics now provide a systems-level view of regulatory networks and metabolism, improving candidate-gene discovery, predictive modelling, and breeding for enhanced grain composition and biofortification (Cai et al., 2023; Mmbando, 2024; Saand and Yang, 2024). Time-course and tissue-specific multi-omics analyses have revealed key genes, TFs, phytohormonal and metabolic pathways controlling seed size, starch and protein content, and responses (Roodt, 2022; Cai et al., 2023; Li et al., 2024d; Yang et al., 2025). These advances illuminate the interconnected gene networks, phytohormone dynamics, and nutrient fluxes that underlie maize seed trait diversity.

### 4.1 Key genes and regulators of maize agronomic seed traits and storage accumulation

Recent omics and co-expression studies have identified key genes and TFs that regulate grain filling and important seed traits, such as size, weight, starch content, and protein composition (**Table 1**). EXPANSIN genes, like *ZmEXPB12* and *ZmEXPB15* are critical for endosperm expansion, enhancing kernel size (Feng et al., 2018; Sun et al., 2022). *ZmMPK6*, a mitogen-activated protein kinase gene, influences starch and protein accumulation (Li et al., 2024b), while *ZmKW1*, an E3 ubiquitin ligase gene, regulates grain-filling, cell size, and cell number within the endosperm in maize kernels (Zhang et al., 2024).

**Table 1:** Key genes and regulators of maize seed traits and compositions.

Gene/Regulator	Role	Citation
ZmNF-YA13	Regulates endosperm cell division and seed size.	(Zhang et al., 2022a)
*ZmSSRP1*	Regulates starch biosynthesis genes.	(Chen et al., 2022)
ZmARF12	Regulates endosperm cell division and enlargement, seed size and weight, and auxin homeostasis.	(Wang et al., 2022)
ZmTCP7	Regulates endosperm starch accumulation.	(Ajayo et al., 2022)
ZmNAC128/ZmNAC130	Regulate starch and protein biosynthesis, zein gene activation.	(Zhang et al., 2019b; Chen et al., 2023; Song et al., 2024)
*ZmHXK3a*	Catalyzes glucose to glucose-6-phosphate for starch metabolism.	(Xiao et al., 2016)
ZmMYB71	Represses starch synthesis genes	(Han et al., 2024)
O11	Activates zein and starch genes, regulates nutrient metabolism	(Feng et al., 2018)
ZmABI19	Regulates zein and starch biosynthesis, embryo development	(Yang et al., 2021a)
*ZmEXPB15*	Promotes nucellus elimination, seed size, and weight	(Sun et al., 2022)
*ZmEXPB12*	Promotes endosperm cell expansion, seed size, and weight.	(Ji et al., 2022)
ZmNAC11 & ZmNAC29	Regulates nucellus degradation, seed size, and weight	(Sun et al., 2022)
ZmMAPK6	Regulates kernel weight, starch content, and grain-filling	(Li et al., 2024b)
*ZmYUC1*	Modulates auxin biosynthesis, seed size, and weight	(Zhang et al., 2022a)
ZmMYB74/ZmMYB138	Regulate endosperm cell division, seed size	(Wang et al., 2023c)
ZmGRAS11	Regulates endosperm cell expansion, seed size and weight.	
ZmPBF1	Regulates γ-zein genes and balances storage protein and starch biosynthesis	(Ning et al., 2023)
O2	Controls endosperm development and storage accumulation by regulating zein biosynthesis, starch synthesis, and cell expansion via interactions with key transcriptional regulators.	(Zhang et al., 2016b; Zhan et al., 2018; Ji et al., 2022; Chen et al., 2023; Wu et al., 2024)
ZmOHP1 & ZmOHP2	Regulates γ-zein genes and interacts with O2.	
ZmbZIP22	Activates 27-kD γ-zein gene expression.	(Li et al., 2018)
ZmNKD1/ZmNKD2	Regulate endosperm development, storage protein and starch biosynthesis	(Wu et al., 2024)
ZmMADS47	Works in synergy with O2 to activate the transcription of zein genes.	(Qiao et al., 2016)
*ZmKW1*	Regulate seed size and weight by influencing endosperm cell number and size.	(Zhang et al., 2024)

Grain filling activates genes involved in DNA replication, metabolite transport, and storage reserve deposition, leading to starch and storage protein accumulation (Li et al., 2023a). Zeins account for ~60% of seed storage proteins, dominated by α-zeins, while γ- and β-zeins provide structural support. Modulating zein expression can alter endosperm texture and nutritional quality (Wu and Messing, 2010; Li and Song, 2020). Starch biosynthesis is driven by enzymes such as AGPase (*ZmBt2*, *ZmSh2*), GBSS1, multiple starch synthases (SS1–SS4), branching enzymes (SBEs), and debranching enzymes (ISA1, ISA2, PUL) (Ajayo et al., 2022; Liu et al., 2022). Additional regulators, including *ZmSSRP1*, hexokinases (e.g., *ZmHXK3a*), and trehalose-6-phosphate (T6P) synthases, coordinate carbon flux into starch biosynthesis (Xiao et al., 2016; Chen et al., 2022; Finegan et al., 2022).

TFs exert central regulatory influence over seed development by either activating or suppressing processes related to cell division, differentiation, nutrient uptake, and storage compound accumulation. Negative regulators such as ZmARF12 suppress seed size (Wang et al., 2022), whereas ZmCYCB1–1 and ZmNF-YA13 enhance endosperm cell proliferation through auxin signaling (Zhang et al., 2022a; Zhao et al., 2022). MYB TFs, such as ZmMYB74 and ZmMYB138, further influence endosperm growth, with loss-of-function mutants resulting in larger kernels (Wang et al., 2023c).

Protein accumulation is largely coordinated by the bZIP TF Opaque2 (O2), which binds α-zein promoters and interacts with partners such as ZmPBF1, ZmbZIP22, ZmOHP1/2, ZmMADS47, and ZmNAC128/130 (Zhang et al., 2015; Yang et al., 2016; Zhang et al., 2016b; Zhan et al., 2018; Cai, 2020; Yang et al., 2022c; Chen et al., 2023). These factors collectively regulate α- and γ-zein expression.

Starch biosynthesis is also tightly regulated by TFs. ZmTCP7 and ZmNAC128/130 modulate *ZmBt2* and AGPase activity (Ajayo et al., 2022; Chen et al., 2023); ZmICE1 regulates *ZmGBSS1* and *ZmSS2a* (Liu et al., 2022; Wang et al., 2024b); ZmbZIP75 activates, while ZmMYB71 represses starch biosynthetic genes (Han et al., 2024; Long et al., 2024). Additional regulators, including ZmDOF36, ZmMADS1, ZmEREB25, and ZmARF27, fine-tune amylose content and endosperm starch biosynthesis (Dong et al., 2019; Wu et al., 2019; Wang et al., 2025).

Importantly, starch and protein pathways are interconnected through TF networks that balance carbon and nitrogen allocation. ZmPBF1 coordinates zein and starch-related genes, including *ZmISA1* and *ZmSBE2b*, in response to nitrogen (N) status (Ning et al., 2023). O2 not only regulates zein synthesis but also activates sucrose and starch metabolism genes (*ZmSh1, ZmSus1/2, ZmPPDK1/2, ZmSS2a, ZmSS3a, ZmISA1*), thereby synchronizing protein and carbohydrate accumulation (Zhang et al., 2016b; Deng et al., 2020). Other factors, like ZmNKD1/2, ZmbZIP29, and ZmABI19, support this balance (Yang et al., 2022c; Wu et al., 2024). Together, these findings highlight an intricate genetic and regulatory framework linking starch and protein metabolism, ensuring optimal kernel development, yield, and nutritional quality.

### 4.2 Gene regulatory networks driving the expression of maize seed traits

**Figure 2:**
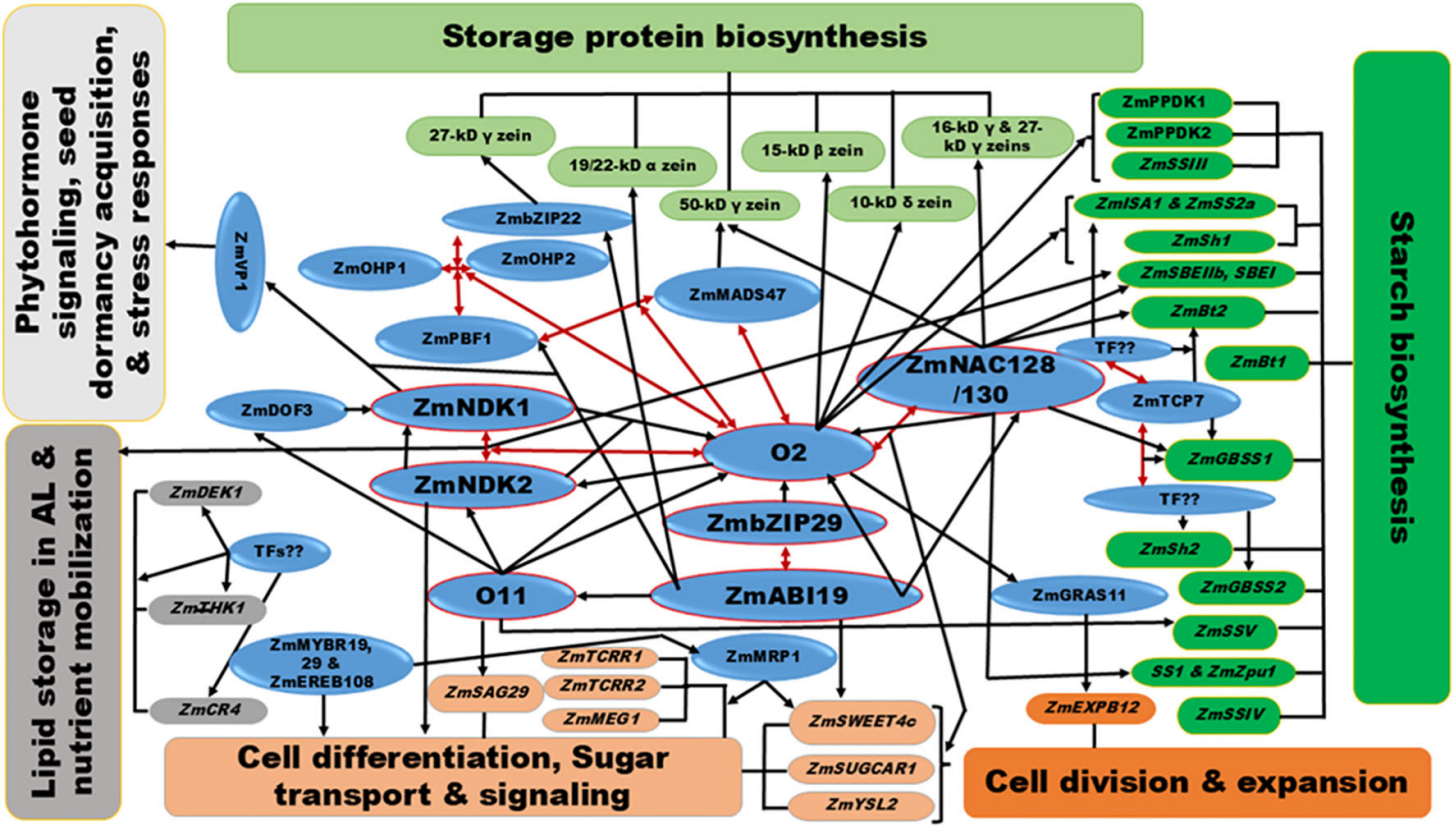
Gene regulatory networks (GRNs) in maize seed development. This figure depicts the gene regulatory networks (GRNs) governing important seed traits in maize. It highlights transcription factors (TFs) in blue ovals as key regulators, illustrating their interactions (double-headed red arrows) and regulation of target genes (single-headed black arrows). Target genes are color-coded by function: starch formation (green), protein biosynthesis (light green), cellular processes (orange), endosperm cell differentiation and sugar transport/signaling (light orange), lipid storage and nutrient mobilization (grey), and phytohormonal signaling/stress responses (light grey). The key regulator Opaque2 (O2) coordinates essential processes like nutrient accumulation by interacting with other critical TFs, including ZmPBF1, ZmOHP1/2, ZmNKD1/2, ZmbZIP22, ZmMADS47, and ZmNAC128/130, which are vital for starch and protein biosynthesis. O2 also establishes feedback loops for cell differentiation and signaling with ZmNKD1/2 and coordinates cell expansion by regulating ZmGRAS11. Other important TFs, such as O11 and emerging regulators like ZmTCP7, ZmMYBR18, and ZmEREB108, further influence grain composition and growth by coordinating sugar transport and signaling and starch deposition. Additionally, ABA-responsive hubs like ZmABI19 and ZmbZIP29 refine these developmental processes by regulating O2 and other key TFs including O11, ZmPBF1, ZmbZIP22, and ZmNAC128/130. Collectively, these networks play a crucial role in determining the composition, quality, and yield of maize seeds.

Understanding the GRNs governing maize seed development is crucial for enhancing grain yield and quality. Recent studies underscore the significant role of TFs within these networks, which orchestrate essential processes for seed development and storage compound accumulation. **Figure 2** depicts these GRNs, highlighting TFs (in blue) linked to target genes involved in various functions: starch formation (green), protein biosynthesis (light green), cellular processes (orange), sugar signaling (light orange), lipid storage (grey), and phytohormonal signaling, desiccation, and stress responses (light grey).

Among these, O2 emerges as a key regulator of grain filling and endosperm development, coordinating protein and starch biosynthesis, cell expansion, and sugar transport through interactions with various TFs (**Figure 2**). Specifically, O2-ZmGRAS11 axis governs endosperm cell expansion and grain filling by modulating *ZmEXPB12* (Ji et al., 2022). Furthermore, O2 establishes a dynamic feedback loop with ZmNKD1/2, impacting their expression through dimerization and reciprocal regulation, which are essential for transcriptional control and chromatin accessibility during maize seed development (Wu et al., 2024). Notably, ZmNKD1 and ZmNKD2 play critical roles in early developmental stages, such as cellularization and aleurone specification, by affecting auxin and ABA signaling via ZmVP1, thereby impacting dormancy, storage reserve accumulation, and stress responses (Gontarek et al., 2016; Zhang et al., 2019a; Zheng et al., 2019; Wu and Becraft, 2021). The regulation of ZmNKD activities is facilitated by ZmDOF3 (Qi et al., 2017).

Moreover, ZmNAC128/130 interacts with O2 to activate starch and γ-zein genes, with knockdowns resulting in severely reduced starch and zein content compared to O2 mutants, underscoring their importance in grain filling. Beyond regulating zein and starch, these NACs also influence nutrient transporters such as *ZmSWEET4c*, *ZmSUGCAR1*, *ZmYSL2*, and are implicated in lipid and auxin biosynthesis, affecting endosperm metabolism and seed quality (Chen et al., 2023; Song et al., 2024). Furthermore, other O2 complexes, including O2-ZmPBF1-ZmOHP1/2 and O2-ZmMADS47, play distinct yet complementary roles in regulating zein gene expression (Zhang et al., 2015; Qiao et al., 2016; Chen et al., 2023).

Another important regulatory hub in maize seed development GRNs is O11, which influences key TFs such as ZmNKD1/2, ZmDOF3, O2, and ZmPBF1. It plays a critical role in storage reserve accumulation, stress responses, BETL differentiation, and sugar signaling, primarily through the target *ZmSAG29* and the dynamic MAPK signaling cascade (Feng et al., 2018). Additionally, emerging regulators like ZmTCP7, ZmEREB108, ZmMYBR19, and ZmMYBR29 significantly contribute to starch biosynthesis and BETL-specific gene activation, affecting seed weight and size and nutrient loading into the endosperm (Ajayo et al., 2022; Yuan et al., 2024).

ABA-responsive regulators ZmbZIP29 and ZmABI19, phosphorylated by ZmSnRK2.2, orchestrate TF cascades linked to storage reserve accumulation and embryo development. They precisely coordinate the activities of key TFs, including O2, O11, ZmbZIP22, ZmPBF1, and ZmNAC128/130, ensuring coordinated gene and developmental processes during seed formation and grain filling (Yang et al., 2021a, c). Collectively, these TFs establish complex gene networks vital for optimal maize seed growth. They include TFs involved in development, carbohydrate and protein metabolism, nitrogen uptake, and phytohormone signaling, making them valuable targets for breeding strategies aimed at improving grain quality, yield, and stress resilience.

Despite notable progress in elucidating the roles of these TFs within regulatory networks governing maize grain filling and development, the pathways involving post-translational modifications of TFs and their impacts on biological functions remain inadequately understood (Yang et al., 2024). Moreover, the analysis of TFs in maize is complicated by functional redundancy and overlapping roles, leading to subtle phenotypes in single mutants (Zhang et al., 2019b; Chen et al., 2023). This intricacy hampers our ability to evaluate their individual contributions. Additionally, the lack of comprehensive spatiotemporal expression profiles for many TFs restricts our understanding of their dynamic functions. While current research has predominantly emphasized endosperm-specific TFs, the influences of TFs on embryo and maternal tissues (seed coat) remain underexplored (Qu et al., 2016; Ajayo et al., 2022; Chen et al., 2023; Song et al., 2024). Finally, the specific interactions and regulatory mechanisms of established TFs in response to phytohormones, such as auxin and ABA, are not fully elucidated (Du et al., 2023).

### 4.3 Phytohormonal crosstalk in maize seed development and storage reserve

Phytohormones like auxins, cytokinins, GA, and ABA play essential roles in maize seed development, influencing important seed traits like size, composition, and grain filling rate and time (Wu et al., 2022; Jain et al., 2023). Cytokinins boost early endosperm formation and cell proliferation, ultimately leading to larger seeds and higher yields (Wu et al., 2022; Jain et al., 2023). However, the intricate biosynthesis and signaling pathways of cytokinins hinder their application in improving crop productivity (Kaur et al., 2022).

Auxin biosynthesis and signaling are also critical for maize development, impacting embryogenesis, endosperm growth, and seed size and weight (Cowling et al., 2023; Du et al., 2023; Song et al., 2024). The auxin response factor ZmARF12, which negatively regulates cell division, shows that its mutants produce larger seeds and heavier grains. Additionally, ZmARF12 works with ZmIAA8 to ensure proper endosperm cell division and seed morphology such as size (Wang et al., 2022). Auxin biosynthesis in maize, occurring via a tryptophan-dependent pathway, involves several genes like ZmYUC1/2/6 and ZmAUX2, all regulated by factors such as ZmNF-YA13, which help control auxin levels (Jiang et al., 2022; Wang et al., 2022; Zhang et al., 2022a). Notably, TFs like ZmNAC128 and ZmNAC130 align auxin biosynthesis with reserve accumulation, thereby integrating phytohormonal signals with metabolic processes crucial for seed formation and filling (Song et al., 2024).

Gibberellin signaling, governed by the GID1–DELLA pathway, is vital for maize seed development, particularly in endosperm cell expansion and storage deposition (Sun, 2010). Mutations in DELLA proteins such as ZmGRAS11 result in smaller seeds due to delayed cell expansion (Ji et al., 2022). Additionally, GA treatments may enhance grain size and bulk density, likely through impacts on cellulose synthesis via TFs like ZmbZIP53 (Lv et al., 2021). ABA is essential for seed maturation, dormancy, grain filling, and nutrient metabolism (Chauffour et al., 2019; Yang et al., 2022c; He et al., 2023). It promotes the phosphorylation of TFs like ZmbZIP75, ZmABI19, and O2, regulating seed dehydration and reserve accumulation (Yang et al., 2022c; Long et al., 2024). However, excessive ABA can prolong dormancy and hinder seed germination, emphasizing the need for balanced phytohormone levels during development (Niu et al., 2022).

These phytohormones operate within a complex, interconnected regulatory network (Jain et al., 2023). Auxin and cytokinin interactions primarily drive cell division and seed growth (Kaur et al., 2022; Wu et al., 2022), while auxin-ABA crosstalk optimizes sink strength and sugar metabolism (Du et al., 2023). Auxin enhances sugar utilization by modulating ABA signaling, upregulating ABA-responsive genes while downregulating ABA biosynthesis, thus reducing seed abortion risks (Du et al., 2023). Moreover, auxin synergizes with GA and ABA to regulate maturation and dormancy and collaborates with JA and brassinosteroids to influence endosperm cell proliferation and differentiation (Matilla, 2020; Wu et al., 2022; Jain et al., 2023). TFs like ZmNKD1/2, O2, and ZmNAC128/130 also interact with auxin and ABA pathways to fine-tune energy distribution and nutrient accumulation (Song et al., 2024; Wu et al., 2024). Overall, these insights highlight phytohormones, especially auxin, as critical integrators of seed developmental, metabolic, and stress signals.

Nevertheless, the precise molecular mechanisms by which phytohormones engage with transcriptional regulators and metabolic enzymes remain unclear. Much of the current understanding is derived from correlative studies or treatments applied externally, which may not truly represent *in vivo* conditions (Du et al., 2023). Furthermore, the variability in phytohormone levels and signaling pathways throughout different tissues of the endosperm is still not well characterized, limiting our grasp of localized phytohormonal regulation (Chen et al., 2024b).

### 4.4 Metabolic pathways conditioning seed filling and quality in maize

#### 4.4.1 Molecular regulation of sucrose transport and signaling in maize grain filling

Maize grain filling is largely influenced by genetic factors that manage metabolic and nutrient pathways. During this critical phase, the regulation of sucrose transport and metabolism is crucial for maximizing yield and seed quality (Braun et al., 2014; Ma et al., 2023). Sucrose, produced in the leaves, is transported to the developing seeds, where it contributes to starch and protein biosynthesis (Shen et al., 2022; Ma et al., 2023). Sucrose transporters play a vital role in moving sucrose between source and sink tissues, ensuring effective carbon allocation (Sosso et al., 2015; Bezrutczyk et al., 2018; Yang et al., 2022a).

The balance between vegetative growth and seed development is essential, particularly during stress, as seeds compete for limited metabolites, which can impact overall yield and quality (Ren et al., 2022). Effective sucrose transport from leaves to seeds is necessary for proper endosperm development and storage reserve accumulation (Sosso et al., 2015; Yang et al., 2022a). Specialized transport proteins, such as SUTs and SWEETs, facilitate phloem loading and unloading, providing critical carbohydrates for grain filling (Sosso et al., 2015; Bezrutczyk et al., 2018; Shen et al., 2022). Key sugar transporter genes, including *ZmSWEET3a/4c/11/13b*, *ZmSUT1*, *ZmSUT4*, ZmYSL2, *ZmSUGCAR1*, *ZmSAG29*, *ZmMN1*, and *ZmSTP3*, are primarily expressed at the maternal-zygotic interface, supporting essential transport processes for endosperm development (Shen et al., 2022; Yang et al., 2022a; Peng et al., 2024). Mutations in transporters like *ZmSUGCAR1* and *ZmSWEET4c* can hinder sugar accumulation, leading to impaired grain filling and defective kernels (Sosso et al., 2015; Yang et al., 2022a).

Molecular mechanisms involving TFs, phytohormonal signals, and metabolic pathways are vital for regulating sucrose transport and signaling during maize grain filling (Yang et al., 2022c; Song et al., 2024). Key TFs such as ZmABI19, ZmNAC128/130, and ZmICE1a influence sugar transporter expression and resource allocation, enhancing sucrose transport and seed development (Yang et al., 2022c; Song et al., 2024; Wang et al., 2024b). This interplay between sucrose transport and phytohormonal regulation creates a complex network essential for seed filling, a subject still underexplored in maize research.

Sucrose signaling closely interacts with metabolic enzymes like sucrose synthase, trehalose- 6-phosphate (T6P), sucrose non-fermenting kinases (suck as SnRK1) and invertases, which are crucial for sucrose metabolism and resource allocation. The T6P-SnRK1 pathway is particularly significant; elevated T6P levels indicate carbon surplus, inhibiting SnRK1 and promoting storage compound biosynthesis. In contrast, low sucrose or stress reduces T6P levels, activating SnRK1 to prioritize energy conservation (Zhang et al., 2009; Bledsoe et al., 2017; Deng et al., 2020; Van Leene et al., 2022). SnRK1 directly regulates starch and protein biosynthesis by phosphorylating TF O2 or the intermediary ZmRFWD3 (Li et al., 2020; Yang et al., 2024). Additionally, SnRK2 phosphorylates factors such as O2, ZmABI19, and ZmbZIP75, linking stress responses to storage metabolism (Yang et al., 2022c; Long et al., 2024). Ultimately, the integration of sucrose transport, metabolism, and energy signaling via the T6P-SnRK1/2 pathway is crucial for optimizing resource use and maintaining energy balance during maize grain filling, thereby enhancing seed size, yield and resilience to stress.

#### 4.4.2 Molecular regulation of nutrient uptake, storage, and bioavailability

Nutrient uptake, transport, storage, and bioavailability significantly influence maize grain composition, yield, and quality, particularly during grain filling. Key macronutrients, such as nitrogen (N), phosphorus (P), and potassium (K), play vital roles in this process (Ocwa et al., 2023). Nitrogen enhances sucrose synthesis and transport to seeds and regulates related enzymes and phytohormones (Guo et al., 2022; Yue et al., 2022). Potassium improves water-use efficiency and aids sucrose synthesis (Gomaa et al., 2021; Fan et al., 2022), while phosphorus supports nutrient remobilization and enhances grain weight (Saeed et al., 2023; Sun et al., 2023).

Genetic and molecular regulation of nutrient dynamics in maize involves TFs, nutrient transporters, and QTLs that affect root development, crucial for nutrient uptake under deficiency. Maize hybrids exhibit heterosis, improving nutrient efficiency in low-input environments (Wu et al., 2021; Li et al., 2024a, c; Peng et al., 2024). Specific QTLs and candidate genes, such as *Zm00001d047728* (*MAPKKK protein kinase*), contribute to P acquisition, while genes like *Zm00001d038281* (*ZmDOF20*) and *Zm00001d052340* (*CBL-interacting kinase*) are linked to N and K metabolism (Tang et al., 2015; Ribeiro et al., 2023; Li et al., 2024a).

Under nutrient stress, maize activates genes for nutrient uptake and response through transcriptional reprogramming. TFs, including ZmMYB62, ZmbZIP11, ZmARF4, ZmARF10, and ZmARF16, promote root development, enhancing nutrient acquisition (Ribeiro et al., 2023; Rajput et al., 2024). In the endosperm, regulators like ZmNAC128/130 and O11 coordinate nutrient storage by targeting transporters (Feng et al., 2018; Peng et al., 2024), and ZmPBF1 balances carbohydrate and protein accumulation based on N availability, ensuring seed quality amid varying nutrient conditions (Ning et al., 2023). Transcriptome analyses of maize under nutrient stress have revealed critical genetic components in N, P, and K metabolism (Singh et al., 2022). Key transporters, such as *ZmNPF1.1*, *ZmPHO1*, and *ZmPHO2*, regulated by ZmNAC128/130, facilitate N and P transfer to developing seeds (Peng et al., 2024). Moreover, metal transporters like *ZmIRT1*, *ZmNAS5*, and *ZmYSL2* enhance zinc (Zn) and iron (Fe) content, boosting seed nutritional quality (Wu et al., 2021; Chao et al., 2023).

To combat malnutrition, maize biofortification efforts aim to increase micronutrient levels, utilizing genetic variations that enhance Zn and Fe in quality protein and provitamin A maize (Chao et al., 2023; Goredema-Matongera et al., 2023). Overexpressing the Zn transporter *ZmYSL2* raises seed Zn levels, while introducing the *lpa1* gene decreases phytic acid, thereby improving Zn and Fe bioavailability (Chao et al., 2023; Yathish et al., 2023). These strategies contribute to developing maize varieties with better nutrient efficiency, quality, and resilience, supporting sustainable agriculture and global food security.

Phytohormones and sugar signaling indirectly influence the expression of nutrient transporter genes through TF networks (Ning et al., 2023; Peng et al., 2024). Research indicates a close relationship between the regulation of transporter genes and the biosynthesis of storage compounds (Hartings et al., 2011; Peng et al., 2024). Importantly, TFs like ZmNAC128/130 also regulate transporter genes related to heavy metals and biofortification, highlighting the intricate coordination necessary for effective seed filling (Peng et al., 2024).

While the regulation of these transporters signals significant progress in understanding nutrient uptake during endosperm filling, the integration of transporter expression with metabolic and phytohormonal signals needs further investigation. Many transporter genes remain functionally uncharacterized, and their expression patterns within the maize kernel require more research. Additionally, understanding the trade-offs between nutrient uptake efficiency and stress responses remains a critical area for future studies (Feng et al., 2018).

#### 4.4.3 Molecular regulation of starch and storage protein biosynthesis pathways

Maize kernels are composed largely of starch (70–75%) and storage proteins (~10%). The coordinated biosynthesis of these reserves in the endosperm is essential for yield and nutritional quality (Ajayo et al., 2022; Chen et al., 2022, 2023; Yang et al., 2022c). This process depends on the integration of TFs, phytohormonal cues, and metabolic regulators, which together optimize gene expression and resource allocation during grain filling (Yang et al., 2022c; Song et al., 2024).

TFs are central to regulating storage reserve genes, while phytohormones provide developmental and environmental signals that modulate their activity (Yang et al., 2022c; Chen et al., 2023; Du et al., 2023; Song et al., 2024). Metabolic regulators, including sugar signaling components and nutrient transporters, further align carbon and nitrogen fluxes (He et al., 2024; Peng et al., 2024). Modern approaches such as ChIP-seq, RNA-seq, and mutant analyses have revealed how TFs and their targets drive reserve accumulation, while single-cell transcriptomics is uncovering cell-type-specific gene expression during endosperm development (Yang et al., 2022c; Song et al., 2024; Wu et al., 2024; Yuan et al., 2024).

Several TFs, including O2, ZmTCP7, ZmNKD1/2, and ZmABI19, play key roles in starch and zein-protein biosynthesis. Their disruption leads to poor kernel filling, reduced starch and protein content, and diminished grain weight, underscoring their importance for sink strength. These regulators control enzymes such as AGPase and starch synthases, as well as nutrient transporters like *ZmSWEET4c* and *ZmYSL2* (Ajayo et al., 2022; Yang et al., 2022c; Chen et al., 2023; Wu et al., 2024).

Network-level regulation ensures robust kernel development. ZmABI19 sits at the core of a transcriptional cascade governing grain filling, while O11 modulates upstream regulators such as O2 and ZmPBF1, integrating environmental signals (**Figure 2**). Mutations in either TF result in smaller, nutrient-deficient kernels (Feng et al., 2018; Yang et al., 2021a, c). Additional layers of control involve feedback loops and post-translational modifications: phosphorylation via SnRK1/2 kinases and ABA-mediated signaling help fine-tune TF activity. TFs such as O2, ZmABI19, ZmbZIP29, and ZmbZIP75 exemplify how regulatory proteins integrate developmental and metabolic signals through phosphorylation-dependent dynamics (Li et al., 2020; Yang et al., 2022c, 2024; Long et al., 2024).

Phytohormones add a further layer of regulation. ABA activates TFs through the SnRK2 pathway, promoting starch and protein biosynthetic genes while linking stress responses with energy allocation (Yang et al., 2022c, 2024; He et al., 2024; Long et al., 2024). Auxin supports sugar metabolism and storage reserve accumulation, often acting synergistically with ABA to coordinate seed filling (Du et al., 2023; Song et al., 2024).

Metabolic regulators, including sugars, kinases, and key biosynthetic enzymes such as AGPase, work alongside TFs and phytohormones to shape reserve depositions. Sucrose and T6P modulate activity of TFs like ZmEREB156 and ZmABI19 to influence expression of biosynthetic genes (Huang et al., 2016; Yang et al., 2022c). Additionally, the kinases such as SnRK1a1 enhance starch biosynthesis by regulating downstream transcriptional programs (Yang et al., 2024). Negative regulators like ZmTCP7 highlight the existence of complex feedback loops, though these remain poorly defined (Ajayo et al., 2022). Similarly, regulation of transporter genes is emerging as a key factor but is not yet fully integrated into broader models of seed filling (Chen et al., 2023; Peng et al., 2024). Although metabolic regulators are integral to transcriptional networks during seed filling. However, research often focuses on these regulators in isolation or within specific developmental stages, restricting our understanding of their broader regulatory interactions and functions (Huang et al., 2016; Yang et al., 2022c, 2024).

A comprehensive, multi-omics approach, combining transcriptomics, genomics, proteomics, metabolomics, and phytohormone profiling, will be crucial for disentangling these interconnected networks. Such integration will clarify how TFs, signaling pathways, and metabolic regulators collectively drive starch-protein coordination, with direct implications for breeding maize varieties with improved yield, resilience, and nutritional quality.

## 5 Conclusions and future perspectives

This review has synthesized the advancements in understanding the genetic, molecular, and metabolic networks that govern key agronomic traits in maize seeds. Advances in high-throughput sequencing, phenotyping, and multi-omics profiling have significantly enhanced our insight into these networks. By integrating data from genomics, transcriptomics, proteomics, metabolomics, researchers have identified crucial regulators and genes involved in seed development and important kernel traits like size, weight, composition, quality, and stress resilience. These methods have improved the discovery of candidate genes and stable QTLs, leading to better predictive models for maize yield and quality. Consequently, these advancements are enabling more informed breeding strategies to improve maize production.

Despite significant advancements, challenges remain in translating seed development into yield in maize. Integrating multi-omics data across various developmental stages, tissues, genotypes, and variable environmental conditions is technically complex, complicating our understanding of the dynamic nature of seed physiology. Issues like dynamic metabolic fluxes, temporal and spatial misalignment of datasets, and underutilization of single-cell and spatial omics limit our capacity to resolve cell type–specific regulation. Moreover, inconsistencies in methods and the interplay of genetic effects continue to constrain breeding model accuracy and translational value. Furthermore, there is a pressing need to establish standardized methodologies for capturing the variability observed in field conditions, ensuring that laboratory findings effectively translate into practical outcomes for maize breeding.

Importantly, a forward-looking perspective highlights several core issues. Climate variability necessitates breeders to prioritize not just yield, but also resilience to environmental stresses like drought and extreme temperatures. Developing climate-adaptive maize cultivars will require integrating genetic and molecular knowledge with advanced breeding techniques such as genome editing and marker-assisted selection. Additionally, as global populations grow and dietary needs change, improving the nutritional quality of maize becomes increasingly important. Addressing malnutrition through biofortification will involve enhancing micronutrient content, which requires in-depth research into the genetic and molecular mechanisms of nutrient uptake and storage. A multidisciplinary approach that includes genetics, agronomy, and nutritional sciences will be vital for this endeavor.

In conclusion, advancing maize breeding to address future food security challenges demands a deep understanding of the biological networks that influence seed traits via thorough functional validation of candidate genes. Future initiatives should emphasize standardized approaches for integrating multi-omics data and enhance the use of single-cell, spatial, and multi-environment omics, alongside gene editing and predictive breeding methods. Combining precision agriculture with innovative breeding technologies will be essential for developing high-yielding, nutritious, and resilient maize varieties. Collaborative efforts among researchers, breeders, and policymakers will be crucial for achieving these goals and ensuring sustainable maize production worldwide.
